# Low protein diet and methyl-donor supplements modify testicular physiology in mice

**DOI:** 10.1530/REP-19-0435

**Published:** 2020-03-10

**Authors:** Hannah L Morgan, Isaac Ampong, Nader Eid, Charlène Rouillon, Helen R Griffiths, Adam J Watkins

**Affiliations:** 1 Division of Child Health, Obstetrics and Gynaecology, Faculty of Medicine, University of Nottingham, Nottingham, UK; 2 Faculty of Health and Medical Sciences, University of Surrey, Stag Hill, Guildford, UK; 3 INRA, Fish Physiology and Genomics, Bat 16A, Campus de Beaulieu, Rennes, France

## Abstract

The link between male diet and sperm quality has received significant investigation. However, the impact diet and dietary supplements have on the testicular environment has been examined to a lesser extent. Here, we establish the impact of a sub-optimal low protein diet (LPD) on testicular morphology, apoptosis and serum fatty acid profiles. Furthermore, we define whether supplementing a LPD with specific methyl donors abrogates any detrimental effects of the LPD. Male C57BL6 mice were fed either a control normal protein diet (NPD; 18% protein; *n* = 8), an isocaloric LPD (LPD; 9% protein; *n* = 8) or an LPD supplemented with methyl donors (MD-LPD; choline chloride, betaine, methionine, folic acid, vitamin B12; *n* = 8) for a minimum of 7 weeks. Analysis of male serum fatty acid profiles by gas chromatography revealed elevated levels of saturated fatty acids and lower levels of mono- and polyunsaturated fatty acids in MD-LPD males when compared to NPD and/or LPD males. Testes of LPD males displayed larger seminiferous tubule cross section area when compared to NPD and MD-LPD males, while MD-LPD tubules displayed a larger luminal area. Furthermore, TUNNEL staining revealed LPD males possessed a reduced number of tubules positive for apoptosis, while gene expression analysis showed MD-LPD testes displayed decreased expression of the pro-apoptotic genes *Bax*, *Csap1* and *Fas* when compared to NPD males. Finally, testes from MD-LPD males displayed a reduced telomere length but increased telomerase activity. These data reveal the significance of sub-optimal nutrition for paternal metabolic and reproductive physiology.

## Introduction

Studies have indicated that male reproductive fitness has declined globally over recent decades (Levine *et al*. 2017). Many factors have been shown to impact negatively on male reproductive fitness and capacity, including obesity (Crean & Senior 2019), smoking (Ranganathan *et al*. 2019), diabetes (Lu *et al*. 2017) and genetic polymorphisms (Louie *et al*. 2016). Of these, the global rise in rates of obesity and metabolic disease have received significant attention (Liu & Ding 2017). Some studies in both humans and animal models have reported negative associations between the consumption of high fat diets or increasing male BMI and sperm quality, embryo development and fetal growth (Bakos *et al*. 2011, Mitchell *et al*. 2011, Colaci *et al*. 2012). However, other studies report only slight increases in rates of oligozoospermia in obese men as compared with men of normal weight (Jensen *et al*. 2004). Similarly, studies in rats have reported that, while prevalence of asthenozoospermia was increased in overweight males, no increase in rates of oligozoospermia were observed (Jia *et al*. 2018). As such, an absolute link between male obesity and reduced sperm quality is still to be defined.

Obesity is characterised by the increased deposition of ectopic adipose tissue (Szendroedi & Roden 2009), elevated levels of plasma fatty acids and triacylglycerol and an increased tendency to develop insulin resistance (Stinkens *et al*. 2015) and metabolic diseases such as type 2 diabetes (Saponaro *et al*. 2015). Links between obesity and male reproductive health are understandable, as cholesterol is the predominant metabolic precursor in the synthesis of testosterone, the main regulator of spermatogenesis. Animal studies have observed links between elevated serum cholesterol, impaired steroidogenesis, testicular morphology and male fertility (Rulli *et al*. 2003, Khorrami *et al*. 2015, Whitfield *et al*. 2015). In addition to the effects on steroidogenesis, plasma fatty acids are also transported around the body via circulation, being taken up and metabolised by numerous tissues resulting in lipotoxicity, tissue inflammation and cell death (Lumeng & Saltiel 2011). Particularly sensitive to the lipotoxic effects of type 2 diabetes or obesity are the testes. As the testes are rich in polyunsaturated fatty acids (PUFAs), they are susceptible to lipid peroxidation in the presence of reactive oxygen species, further enhancing lipotoxicity (Asadi *et al*. 2017). In rodents, the hyperglycaemia, hyperinsulinemia and hypercholesterolemia associated with high fat diets correlate with significant changes in testicular morphology (Campos-Silva *et al*. 2015, Fan *et al*. 2015). Furthermore, animal studies identify increased levels of reactive oxygen species and apoptosis of seminiferous tubule germ cells as mechanisms linking dietary fat intake with altered testicular morphology (Ghosh & Mukherjee 2018, Simon *et al*. 2018). However, in humans, clear links between serum lipid profiles and male fertility remain to be defined with some studies showing a negative impact of elevated cholesterol and phospholipids (Schisterman *et al*. 2014) while others do not (Hagiuda *et al*. 2014). Therefore, changes in paternal nutrition and physiology could alter circulating lipid profiles which then impact directly or indirectly on testicular cell death, seminiferous tubule morphology and ultimately reproductive fitness (Oliveira *et al*. 2017).

There is also growing evidence that poor diet elevates levels of serum and tissue homocysteine, perturbing cellular 1-carbon metabolism, impacting negatively on male fertility (Singh & Jaiswal 2013). Central to the 1-carbon metabolism cycle is the re-methylation of homocysteine to methionine by methionine synthase. This reaction prevents the cellular build-up of homocysteine and ensures an adequate supply of methionine for the synthesis of S-adenosylmethionine, an essential cofactor in the methylation of DNA and histones (Ducker & Rabinowitz 2017). Changes in DNA methylation profiles have been identified in sperm of infertile men (Sujit *et al*. 2018), bulls (Kropp *et al*. 2017), boars (Congras *et al*. 2014) and rats (Song & Yang 2018). Furthermore, studies have shown that, in sub-fertile men, folate supplementation can have beneficial effects on sperm quality (Irani *et al*. 2017). Therefore, dietary induced disturbances in testicular 1-carbon methyl donor metabolism may provide a link between poor paternal diet, sperm epigenetic status and offspring development.

We have shown that offspring of male mice fed a low protein diet (LPD) are born heavier and display impaired glucose metabolism, increased adiposity and perturbed cardiovascular function in adult life (Watkins & Sinclair 2014, Watkins *et al*. 2018). Furthermore, we observe that sperm from LPD males display genome-wide sperm DNA hypomethylation, associated with changes in testicular expression of central regulators of DNA methylation and 1-carbon metabolism (Watkins *et al*. 2018). However, we observe no detrimental impact of LPD on paternal reproductive fitness as measured by changes in total sperm production, capacity to mate females or litter sizes (Watkins & Sinclair 2014, Watkins *et al*. 2017, 2018). This suggests that paternal LPD impacts more on testicular function and sperm epigenetic status than on sperm production and fundamental fertility. Therefore, in the present study, we define the impact of paternal LPD on testicular morphology and cellular proliferation. Furthermore, we investigate whether supplementing the LPD with vitamins and methyl donors (e.g. vitamin B6, folate, methionine) can negate the detrimental effects of LPD.

## Materials and methods

### Dietary regimens

All experimental and study procedures were conducted under the UK Home Office Animal (Scientific Procedures) Act 1986 Amendment Regulations 2012, which transposed Directive 2010/63/EU into UK law, and were approved by the Animal Welfare and Ethical Review Board at Aston University. Eight-week-old C57BL/6 male mice (Harlan Ltd, Belton, Leicestershire, UK) were maintained and fed either control normal protein diet (NPD; 18% casein; *n* = 8), isocaloric low protein diet (LPD; 9% casein; *n* = 8) or LPD supplemented with 1-carbon methyl donors (MD-LPD; 5 g/kg diet choline chloride, 15 g/kg diet betaine, 7.5 g/kg diet methionine, 15 mg/kg diet folic acid, 1.5 mg/kg diet vitamin B12; *n* = 8). Diets were manufactured commercially (Special Dietary Services Ltd; UK) and their composition is provided in Table 1. After 7 weeks on the respective diets, males were mated to virgin, chow-fed 8-week-old C57BL/6 females (Harlan Ltd, Belton, Leicestershire, UK) with each male mating one single female. Following mating, males were killed via cervical dislocation. Blood samples were taken via heart puncture, centrifuged at 10,600 ***g*** (4°C, 10 min) and the serum aliquoted and stored at −80°C. Testes were dissected and weighted prior to being snap frozen and stored at −80°C (right testis) or fixed overnight in 10% neutral buffered formalin at 4°C prior to wax embedding (left testis). Pregnant females were culled via cervical dislocation for the analysis of litter size and fetal weight.

**Table 1:** Gross composition of the diets used in this study.

Dietary supplement	NPD (g/kg)	LPD (g/kg)	MD-LPD (g/kg)
Casein	180	90	90
Corn Oil	100	100	100
Starch Maize	425	485	485
Cellulose	50	50	50
Sucrose	213	243	243
Vitamins (AIN76)	5	5	5
Minerals (AIN76)	20	20	20
Choline Chloride	2	2	7
D,L-Methionine	5	5	12.5
Betaine	–	–	15
Folic Acid	–	–	0.015
Vitamin B12	–	–	0.0015

### Serum free fatty acid and testosterone analysis

Serum non-esterified fatty acid profiles were determined using a methodology adapted from Ichihara and Fukubayashi (2010). Briefly, an internal standard (Undecanoic acid; C11:0) (Sigma) was added to 50 μL of mouse serum prior to being diluted to a final volume of 450 μL in PBS (Thermo-Fisher). Fatty acids were extracted using a chloroform-methanol mix (2:1; Thermo-Fisher) in 0.01% butylated hydroxytoluene (Sigma) prior to centrifugation at 200 ***g*** for 10 min at 4°C. The organic (chloroform) phase was removed and dried under nitrogen gas. The isolated fatty acids were methylated (200 μLtoluene, 0.3 mL (6.3%) HCL)) in 1.5 mL methanol (all from Thermo-Fisher) at 100°C for 1 h in PTFE-sealed glass vials. The fatty acid methyl esters (FAMEs) were subsequently extracted with 1 mL of hexane (Thermo-Fisher) and 1 mL of water, evaporated under nitrogen and re-suspended in 20 μL of hexane prior to analyses by gas chromatography (7820A (G4350A) GC system; Agilent technologies) equipped with a flame-ionization detector and using an Omegawax 250 capillary column (30 m × 0.25 mm ID × 0.25 µm film; Sigma). Recovery rates were calculated following analysis of serum spiked with undecanoic acid (C11:0, 0.2 mg/mL) in two separate samples. The concentrations of fatty acids in the non-spiked samples were subtracted from the spiked samples and the recovery percentages calculated, observing an average recovery rate of 70.78%. Identification of the fatty acid peaks were determined against peak areas of a Supelco 37 fatty acid methyl ester standard mix (Sigma), and individual fatty acid concentrations were determined by reference to the peak of the internal standard.

Serum testosterone levels were determined using a testosterone ELISA kit (Abcam, #ab108666) according to the manufacturer’s instructions.

### Testicular histology

Wax embedded testes were sectioned at 5 µm using a Leitz 1512 rotary microtome (Leica). For analysis of seminiferous tubule morphology, sections were processed and stained with hematoxylin and eosin prior to imaging using a Leica DMRB microscope and image analysis using ImageJ software. Tubules were analysed for total tubule cross-section area, tubule perimeter, area of the tubule epithelium and area of the tubule lumen. On average, 30–40 individual seminiferous tubules were analysed per male.

For analysis of apoptosis, terminal deoxynucleotidyl transferase (TdT) dUTP Nick-End Labelling (TUNEL) staining was performed on separate testicular sections using the In Situ Cell Death Detection Kit (Roche) according to the manufacturer’s instructions. Briefly, slides were dewaxed and re-hydrated prior to microwave antigen retrieval for 5 min in 0.1 M tri-sodium citrate buffer (pH 6.0). Slides were stained using the supplied TUNEL reaction mixture and incubated for 60 min at 37°C in a humidified chamber and kept in darkness. Sections were mounted in Vectashield (Vector Laboratories, UK) prior to image analysis. Negative (PBS only) and positive (DNAse treated prior to application of supplied TUNEL reaction mixture) slides were prepared and treated in parallel to experimental slides. Sections were imaged using an ECLIPSE 90i (Nikon) microscope. Individual tubules were scored as either containing or lacking any apoptotic cells. On average, 40–50 tubules were analysed per male.

For analysis of Ki67 staining, testicular sections were dewaxed, re-hydrated and microwaved in 0.1M tri-sodium citrate buffer (pH 6.0) for 5 min. Sections were blocked in 10% normal donkey serum (Sigma) diluted in PBS with 1% BSA (Sigma) at room temperature for 1 hour prior to the application of the anti Ki67 antibody (Abcam, catalogue # ab155580) diluted 1:50 in PBS with 1% BSA and incubated at 4°C overnight. Negative controls were incubated in PBS with 1% BSA alone. Slides were washed (three time in PBS) prior to the addition of a fluorescent secondary antibody (donkey anti rabbit IgG, 1:100, Invitrogen) at room temperature for 1 hour. Slides were washed (three time in PBS), mounted in Vectashield (Vector Laboratories, UK) and imaged using an ECLIPSE 90i (Nikon) microscope. On average, 40–50 tubules were analysed per male.

### Testicular and hepatic RNA extraction and gene expression analysis

RNA was extracted from livers and testes using the TissueLyser (Qiagen; 60 s at 25 Hz) and the RNeasy Mini Plus Kit (Qiagen) and quantified by Nanodrop prior to cDNA synthesis using the NanoScript (PrimerDesign, UK) kit, all according to the manufacturer’s instructions. For Real-Time PCR (RT-qPCR), 5 ng cDNA was added to a reaction mixture containing 1X Precision SYBRgreen Mastermix (Primerdesign, UK), 175 nM forward and reverse primers (Eurofins) and water. Amplification and detection of hepatic genes was performed using a Stratagene MX 3000P System (Agilent Technologies), while testicular gene expression was analysed using an Applied Biosystems 7500 Fast system. For both tissues, a post-amplification melting curve confirmed the presence of specific products for each primer set. Ct values were converted to relative expression values using the delta-delta Ct method with gene-of-interest expression normalised to *Pgk1* and *Tbp* for hepatic tissue and *Sdha* and *Tbp* for testicular tissue, both using geNorm software as described previously (Lucas *et al*. 2011). Primer sequences are provided in Table 2.

**Table 2:** List of primers used for RT-qPCR studies.

Gene name	Gene symbol	Accession number	Primer sequences		Amplicon length
			Forward primer	Reverse primer	
Phosphoglycerate kinase 1	*Pgk1*	NM_008828	tacctgctggctggatggaagacc	cacagcctcggcatatttct	65
Succinate dehydrogenase complex, subunit A, flavoprotein	*Sdha*	NM_023281	tgttcagttccaccccaca	tctccacgacacccttctgt	66
TATA box binding protein	*Tbp*	NM_013684.3	gggagaatcatggaccagaa	gatgggaattccaggagtca	90
BCL2-associated agonist of cell death	*Bad*	NM_007522.3	gccctaggcttgaggaagtc	catactctgggctgctggtc	90
BCL2-associated X protein	*Bax*	NM_007527.3	agtgtctccggcgaattg	ccacgtcagcaatcatcct	69
B cell leukemia/lymphoma 2	*Bcl2*	NM_009741.5	gtacctgaaccggcatctg	gctgagcagggtcttcagag	130
Caspase 1	*Casp1*	NM_009807.2	caagttgacctcagagaaatgaag	ggcagcaaattctttcacct	114
Fatty acid binding protein 1, liver	*Fabp1*	NM_017399.4	acttctccggcaagtaccaa	ttccctttctggatgaggtc	214
Fatty acid binding protein 3, liver	*Fabp3*	NM_010174.1	ctttgtcggtacctggaagc	cagagcgctggtcatgtagt	222
Fatty acid desaturase 2	*Fads2*	NM_019699.1	attcgggagaagatgctacg	aagaacttgcccacgaagtc	233
Fas (TNF receptor superfamily member 6)	*Fas*	NM_007987.2	caagtgcaagtgcaaaccag	gggttccatgttcacacga	86
Stearoyl-Coenzyme A desaturase 1	*Scd1*	NM_009127.4	ttccctcctgcaagctctac	cagagcgctggtcatgtagt	156
Bromodomain, testis-specific	*Brdt*	NM_054054.2	agggtgatcggataaggccc	caaccacttcggatcctggt	221
GATA binding protein 1	*Gata1*	NM_008089.2	catcaacaagcccaggttcaa	cagaatccacaaactggggc	98
IQ Motif Containing G	*Iqcg*	NM_178378.3	tggaggagattgagaaactgag	gccaggtcttgcaggtgtac	214
SRY (sex determining region Y)-box 9	*Sox9*	NM_011448.4	gtacccgcatctgcacaac	ctcctccacgaagggtctct	94
TATA-Box Binding Protein Associated Factor 2	*Taf2*	NM_001081288.1	gagtatggcagagagaggtgct	acggatagcgacaagtcaaaat	398
Testis Expressed 101	*Tex101*	NM_019981.2	caggtcttgatcggctcttc	gcaaagttctcctggattgc	214

### Testicular telomere length and telomerase activity analyses

For analysis of testicular telomere length, a real-time PCR (RT-qPCR) method was used to measure relative telomere length against the single copy gene Rplp0 (36B4) as an internal control, using genomic DNA as a template. DNA was extracted from testes using the TissueLyser (Qiagen; 60 ss at 25 Hz) and the DNeasy Mini Kit (Qiagen) according to manufacturer’s instructions. Fifty nanogram of testicular DNA was added to 1X Precision SYBRgreen Mastermix (Primerdesign, UK), 350 nM forward and reverse primers (Eurofins) and water. Amplification and detection was performed using an Applied Biosystems 7500 Fast system. Primer sequences are provided in Table 2.

Paternal testicular tissue telomerase activity was determined using the TeloTAGGG™ Telomerase PCR ELISA (Sigma) according to the manufacturer’s instructions. Briefly, 40–100 mg of testis tissue were lysed using the TissueLyser (90 s at 30 Hz; Qiagen). Samples were incubated on ice for 30 min prior to centrifugation at 16,000 ***g*** for 20 min at 4°C. Protein content of the supernatant was determined by DC protein assay (BioRad) and a total protein extract of 50 µg was used in each reaction assay. All samples were analysed in duplicate and a relative change in telomerase activity was determine at an absorbance of 450 nm.

### Statistical analysis

All data were analysed using GraphPad Prism (version 7). Data were assessed initially for normality (Shaprio–Wilk and Kolmogorov–Smirnov tests) prior to analysis using one-way ANOVA followed by Bonferroni post-hoc test, or Kruskal–Wallis test with Dunns multiple comparison test where appropriate (GraphPad Prism, version 7). Correlations between parameters were conducted using Pearson correlation. Significance was taken at *P* < 0.05.

## Results

**Figure 1:**
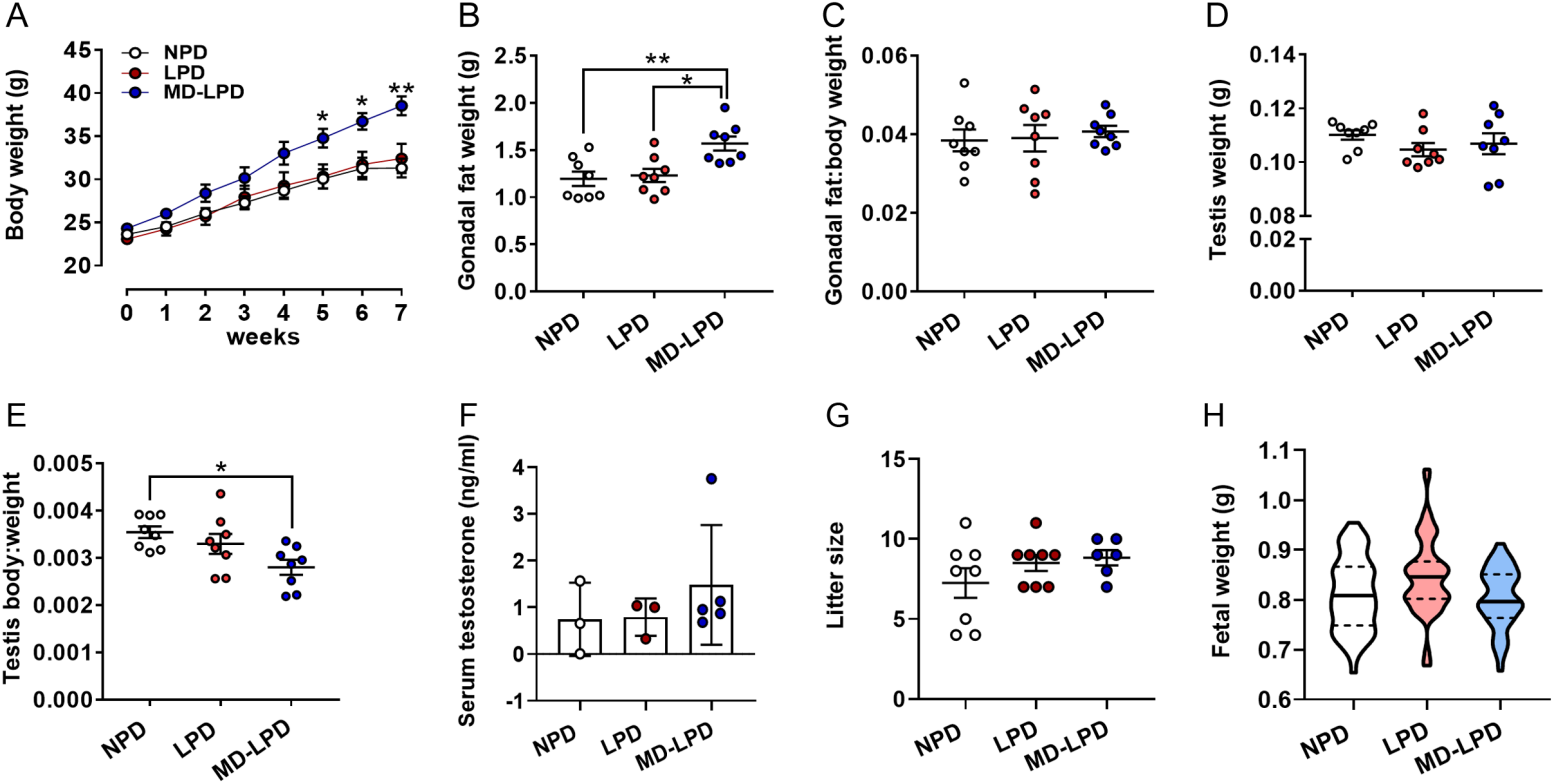
Impact of NPD, LPD and MD-LPD on male growth (A), gonadal fat weight (B) gonadal fat:body weight ratio (C), testis weight (D) testis:body weight ratio (E), serum testosterone (F), late gestation litter size (G) and fetal weight. Data are mean ± s.e.m. in A–G and mean (solid line) with 25 and 75% quartiles (dashed lines) in H. *n* = 8 males per dietary group in A–G and eight litters (each from separate males) representing an *n* of 52–59 fetuses per treatment group in H. Data were analysed by one-way ANOVA followed by Bonferroni post-hoc test, or Kruskal–Wallis test with Dunns multiple comparison test where appropriate. *P < 0.05, **P < 0.01, ***P < 0.001.

After 5 weeks on respective diets, males fed MD-LPD became significantly heavier than males fed either NPD or LPD (Fig. 1A, *P* < 0.05). This increase in body weight coincided with an increase in gonadal fat pad weight in MD-LPD males when compared to NPD and LPD males (Fig. 1B, *P* < 0.05). However, this did not represent a change in gonadal fat pad:body weight ratio (Fig. 1C). No difference in mean testicular weight was observed between groups (Fig. 1D). However, due to the increased body weight observed in MD-LPD males, when expressed relative to body weight, MD-LPD males displayed a lower testis:body weight ratio when compared to NPD males (Fig. 1E, *P* = 0.02). No significant difference in serum testosterone levels were observed between any of the treatment groups (Fig. 1F). Analysis of late gestation litter sizes (Fig. 1G) and fetal weight (Fig. 1H) showed there to be no significant differences between any of the treatment groups.

**Figure 2:**
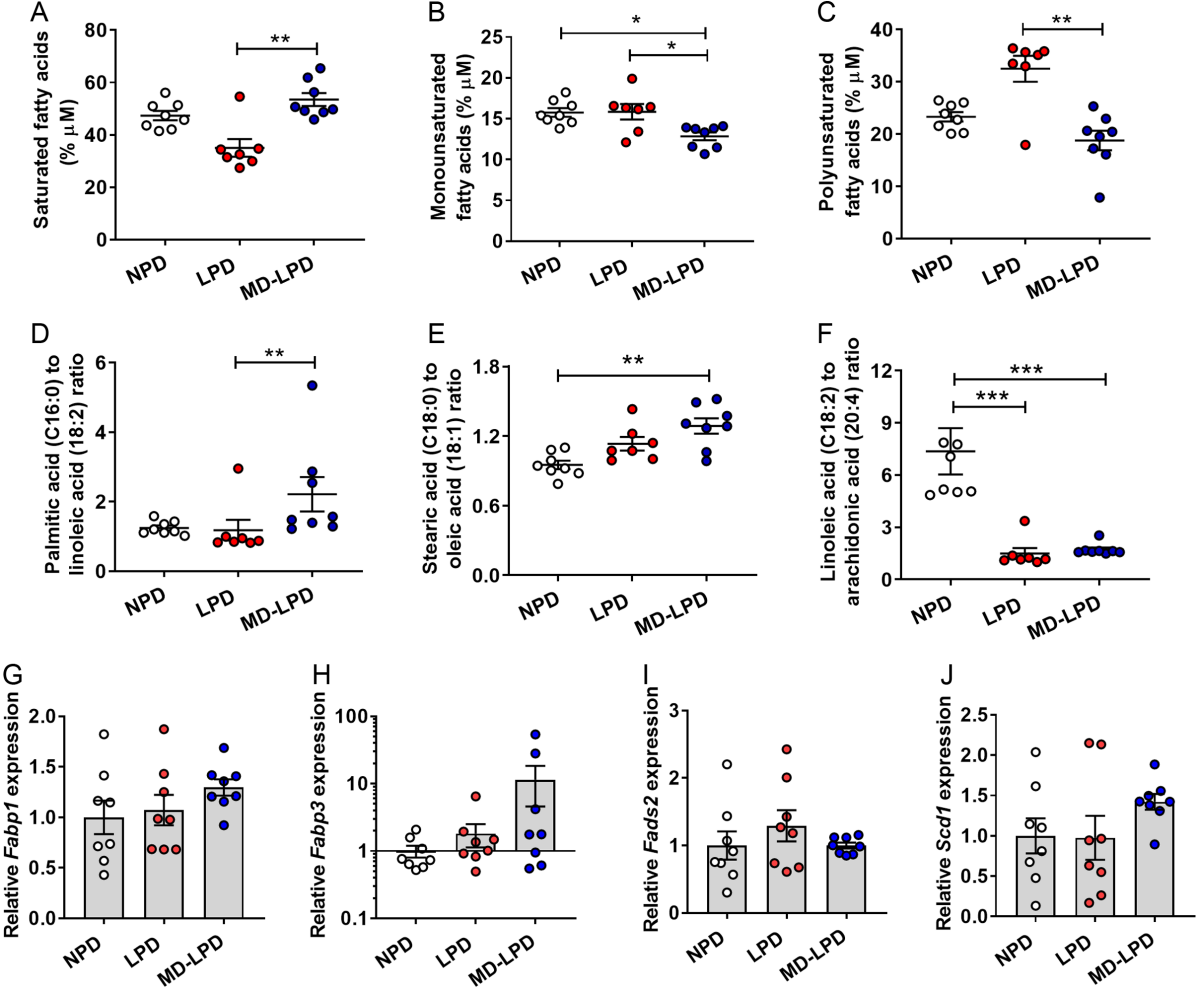
Impact of NPD, LPD and MD-LPD on serum saturated fatty acids (A), monounsaturated fatty acids (B), polyunsaturated fatty acids (C), palmitic:linoleic acid ratio (D), stearic:oleic acid ratio (E) and linoleic:arachidonic acid ratio (F). Relative hepatic expression of fatty acid binding protein 1 (*Fabp1*) (G), fatty acid binding protein 3 (*Fabp3*) (H), fatty acid desaturase 2 (*Fads2*) (I) and stearoyl-Coenzyme A desaturase 1 (*Scd1*) (J). Data are mean ± s.e.m. *n* = 8 males per dietary group. Data were analysed by one-way ANOVA followed by Bonferroni post-hoc test, or Kruskal–Wallis test with Dunns multiple comparison test where appropriate. *P < 0.05, **P < 0.01, ***P < 0.001.

Analysis of serum fatty acid profiles revealed that MD-LPD males displayed elevated levels of saturated fatty acids when compared to LPD males (Fig. 2A, *P* = 0.004), lower levels of monounsaturated fatty acids when compared to NPD (Fig. 2B, *P* = 0.014) and LPD males (Fig. 2B, *P* = 0.039) and lower levels of polyunsaturated fatty acids when compared to LPD males (Fig. 2C, *P* = 0.004). Analysis of individual fatty acids (Table 3) revealed LPD fed males displayed significantly lower proportions of capric acid (C10:0), myristic acid (C14:0) cis-10-Pentadecenoic acid (C15:1), palmitic acid (C16:0) and heptadecanoic acid (C17:0) when compared to NPD and/or MD-LPD fed males (*P* < 0.05), but higher amounts of stearic acid (C18:0), arachidonic acid (C20:4) and lignoceric acid (C24:0) (*P* < 0.05). In addition, MD-LPD males displayed elevated levels of lauric acid (C12:0), heptadecanoic acid (C17:0) and docosanoic acid (C22:0) when compared NPD and/or LPD males (*P* < 0.05), while levels of oleic acid (C18:1), linoleic acid (C18:2) and cis-13,16-docosadienoic acid (C22:2) acid were lower when compared to NPD and/or LPD males (*P* < 0.05). No differences in serum profiles of pentadecanoic acid (C15:0) were observed between groups. Analysis of the palmitic acid (C16:0) to linoleic acid (18:2) ratio (an indicator of *de novo* lipogenesis) revealed an elevated ratio in MD-LPD males which was significant when compared to LPD males (Fig. 2D, *P* = 0.004). Similarly, the ratio of stearic acid (C18:0) to oleic acid (C18:1) was also increased in MD-LPD males when compared to NPD males (Fig. 2E, *P* = 0.005), while the ratio of linoleic acid (C18:2) to arachidonic acid (C20:4) was significantly decreased in both LPD and MD-LPD males when compared NPD males (Fig. 2F, *P* < 0.001).

**Table 3:** Serum fatty acid profiles.

Fatty acid (abbreviated structure)	Diet		
	NPD (% µM)	LPD (% µM)	MD-LPD (% µM)
Capric acid (C10:0)	6.16 ± 0.56^a^	2.68 ± 0.25^b^	6.88 ± 0.50^a^
Lauric acid (C12:0)	3.80 ± 0.60^a^	1.93 ± 0.25^a^	7.15 ± 0.73^b^
Myristc acid (C14:0)	5.19 ± 0.28^a^	2.02 ± 0.14^b^	5.61 ± 0.22^a^
Pentadecanoic acid (C15:0)	1.74 ±0.27^a^	1.31 ± 0.30^a^	1.95 ± 0.21^a^
cis-10-Pentadecenoic acid (C15:1)	1.54 ± 0.21^a^	0.92 ± 0.10^b^	1.22 ± 0.09^a,b^
Palmitic acid (C16:0)	22.94 ± 0.55^a^	19.09 ± 2.76^b^	21.25 ± 1.85^a,b^
Heptadecanoic acid (C17:0)	5.84 ± 0.53^a^	3.03 ± 0.42^b^	8.08 ± 0.44^c^
Stearic acid (C18:0)	13.57 ± 0.73^a^	16.61 ± 0.54^b^	14.90 ± 0.83^a,b^
Oleic acid (C18:1)	14.23 ± 0.42^a^	14.93 ± 1.01^a^	11.62 ± 0.46^b^
Linoleic acid (C18:2)	18.72 ± 0.77^a^	17.60 ± 0.99^a^	11.13 ± 1.03^b^
Arachidonic acid (C20:4)	2.89 ± 0.31^a^	14.08 ± 1.81^b^	6.83 ± 0.78^a,b^
Docosanoic acid (C22:0)	0.73 ± 0.09^a,b^	0.58 ± 0.17^a^	1.68 ± 0.40^b^
cis-13,16-docosadienoic acid (C22:2)	1.71 ± 0.28^a^	0.82 ± 0.27^a,b^	0.81 ± 0.14^b^
Lignoceric acid (C24:0)	0.94 ± 0.21^a^	4.40 ± 0.41^b^	0.89 ± 0.17^a^

Data are mean ± s.e.m. *n* = 8 males per dietary group. Data were analysed by one-way ANOVA followed by Bonferroni post-hoc test, or Kruskal–Wallis test with Dunns multiple comparison test where appropriate. All differences (as denoted by a different subscript letter) represent statistical significance at *P* < 0.05.

To determine if changes in serum fatty acid profiles were underlined by changes in hepatic cholesterol uptake and fatty acid synthesis, we analysed the hepatic transcript expression in our dietary manipulated males. We observed no difference in the relative expression of the fatty acid-binding protein 1 (liver) (*Fabp1*, Fig. 2G), fatty acid-binding protein 3 (heart) (*Fabp3*, Fig. 2H), fatty acid desaturase 2 (*Fads2*, Fig. 2I) or stearoyl-Coenzyme A desaturase 1 (*Scd1,* Fig. 2J) between groups.

**Figure 3:**
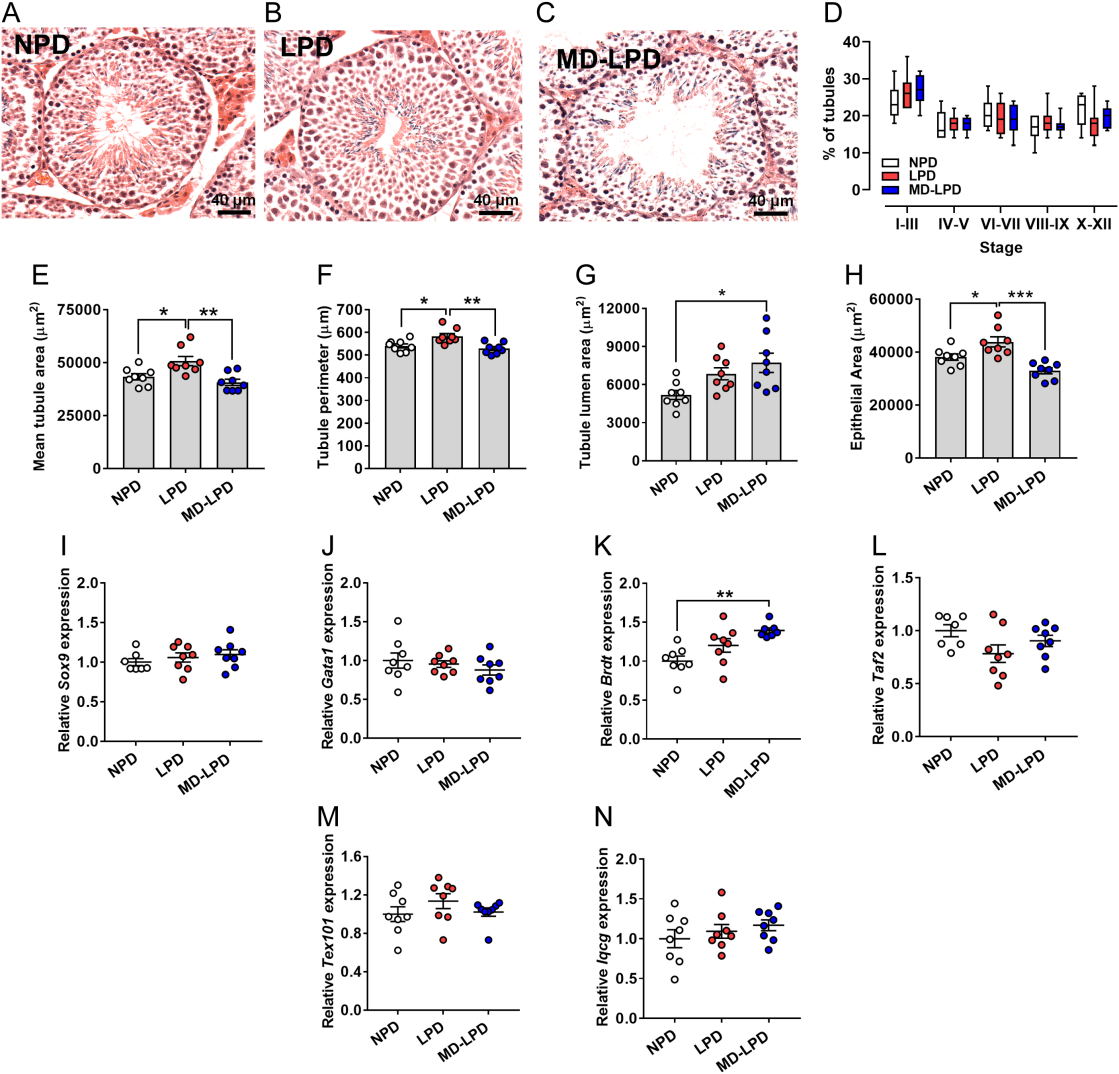
Impact of NPD, LPD and MD-LPD on testicular morphology. Representative cross section image of NPD (A), LPD (B) and MD-LPD (C) seminiferous tubule morphology. Quantification of seminiferous tubule stage in NPD, LPD and MD-LPD testes (D). Mean seminiferous tubule cross section area (E), perimeter (F), lumen area (G) and area of the epithelium (H). Relative testicular expression of SRY sex determining region Y-box 9 (*Sox9*) (I), GATA binding protein 1(*Gata1*) (J), bromodomain, testis-specific (*Brdt*) (K), TATA-Box Binding Protein Associated Factor 2 (*Taf2*) (L), testis Expressed 101 (*Tex101*) (M) and IQ Motif Containing G (Iqcg) (*n*). Data are mean ± s.e.m. *n* = 8 males per dietary group with a minimum of 50 tubules per testis analysed in A–H. Data were analysed by one-way ANOVA followed by Bonferroni post-hoc test, or Kruskal–Wallis test with Dunns multiple comparison test where appropriate. *P < 0.05, **P < 0.01.

We observed significant changes in gross testicular seminiferous tubule morphology in response to diet (Fig. 3A, B and C). While there were no differences in the relative proportions of tubules at each stage of the seminiferous tubule cycle between treatment groups (Fig. 3D), we observed increased mean seminiferous tubule cross-section area (Fig. 3E), perimeter (Fig. 3F) and area of epithelium (Fig. 3H) in testes from LPD fed males as compared to NPD and MD-LPD males (*P* < 0.05). In addition, testes from MD-LPD males displayed increased cross-section luminal area when compared to NPD males (Fig. 3G, *P* = 0.011). Expression analysis of multiple seminiferous tubule cell-specific markers revealed no difference in levels of the sertoli cell markers *Sox9* or *Gata1* (Fig. 3I and J), the spermatocyte and spermatid markers *Taf2* and *Tex101* (Fig. 3L and M) or the maturing spermatid marker *Iqcg* (Fig. 3N). However, increased expression of the pachytene and diplotene spermatocyte marker *Brdt* was observed in MD-LPD testes when compared to NPD testes (*P* < 0.01; Fig. 3K).

**Figure 4:**
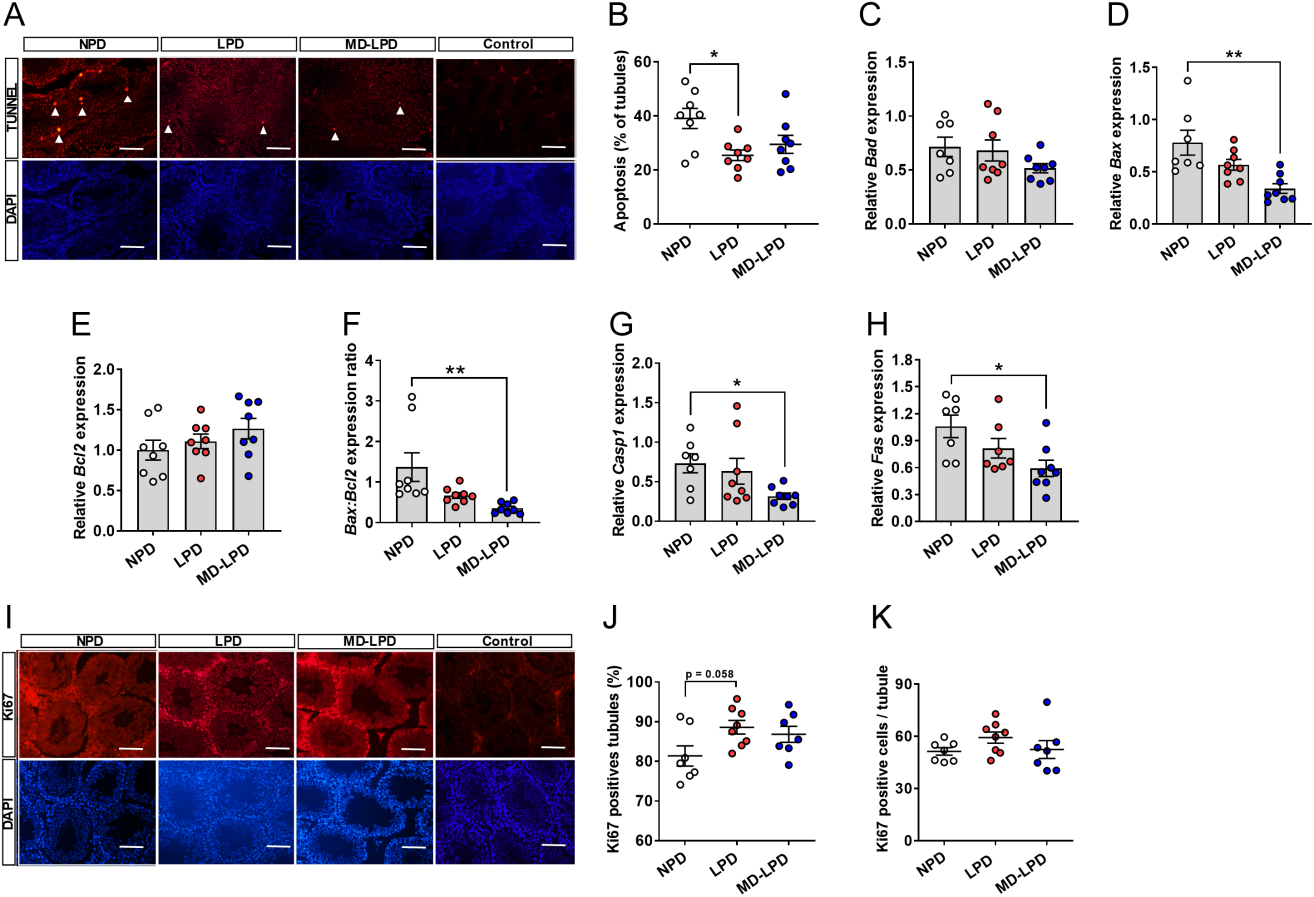
Impact of NPD, LPD and MD-LPD on testicular apoptosis. Representative TUNEL and DAPI stained sections of NPD, LPD and MD-LPD testis and negative control (bar = 100 µm) (A) showing apoptotic cells (white arrow heads) and the percentage of tubules showing apoptotic cells (B). Relative testicular expression of BCL2-associated agonist of cell death (*Bad*) (C), BCL2-associated X protein (*Bax*) (D), B cell leukemia/lymphoma (*Bcl2*) (E), *Bax:Bcl2* expression ratio (F), caspase 1 (*Casp1*) (G) and TNF receptor superfamily member 6 (*Fas*) (H). Representative Ki67 and DAPI stained sections of NPD, LPD and MD-LPD testis and negative control (bar = 100 µm) (I) with the percentage of tubules showing positive stained cells (J) and mean number of Ki67 positive cells per tubule (K). Data are mean ± s.e.m. *n* = 8 males per dietary group. Data were analysed by one-way ANOVA followed by Bonferroni post-hoc test, or Kruskal–Wallis test with Dunns multiple comparison test where appropriate. *P < 0.05, **P < 0.01.

To determine the mechanisms underlying the increase in tubule cross-section area, we analysed testicular profiles of apoptosis and proliferation. We observed decreased proportions of tubules with TUNNEL positive cells in the testes of LPD males when compared to NPD males (Fig. 4B; *P* = 0.014). Expression analysis of central regulators of apoptosis revealed decreased expression of *Bax, Casp1* and *Fas* in the testes of MD-LPD males when compared to NPD males (Fig. 4D, G and H). No differences in the expression of *Bad* or* Bcl2* were observed between treatment groups (Fig. 4C and E). We also observed a significant reduction in the relative *Bax:Bcl2* ratio between NPD and MD-LPD males (Fig. 4F, *P* = 0.007). While this ratio was also reduced in the LPD males, it did not reach significance when compared to NPD males (*P* = 0.07). In contrast, we observed increased number of tubules staining positive for the marker of proliferation, Ki67 in LPD tubules when compared to NPD tubules (Fig. 4J). However, there were no differences in the mean number of Ki67 positive cells per tubule between groups (Fig. 4K).

**Figure 5:**
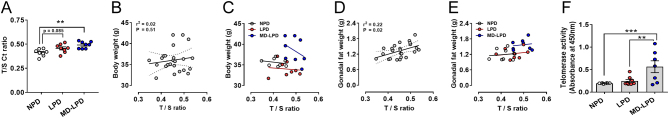
Impact of NPD, LPD and MD-LPD on testicular telomere length (as determined by the T/S ratio) (A). Correlation of body weight to T/S ratio in all males (B) and individual dietary groups (C). Correlation of gonadal fat weight to T/S ratio in all males (D) and individual dietary groups (E). Testicular telomerase activity (F). Data are mean ± s.e.m. *n* = 8 males per dietary group. Data were analysed by one-way ANOVA followed by Bonferroni post-hoc test, or Kruskal–Wallis test with Dunns multiple comparison test where appropriate. Correlations were analysed using Pearsons correlation. **P < 0.01, ***P < 0.001.

To assess whether telomere length was related to the observed changes in testicular morphology and cellular proliferation, we measured testicular telomere length by RT-qPCR. We observed that, in the testes of MD-LPD fed males, the telomere to single copy gene ratio (ratio of the cT for telomere and the single copy gene) was significantly increased (indicating shorter telomere length) when compared to NPD males (Fig. 5A; *P* = 0.002). To define the associations between testicular telomere length and male physiology further, we correlated telomere length (as defined by the T/S ratio) with body weight and gonadal fat weight. We observed no significant correlation between telomere length and body weight either when all males were analysed together or as individual diet groups (Fig. 5B and C). However, in all groups, a significant correlation between telomere length and gonadal fat mass was observed (Fig. 5D; *P* = 0.02), with a significantly elevated level of association observed in MD-LPD males when compared to both NPD and LPD males (Fig. 5E; *P* = 0.035). Finally, we determined the activity of testicular telomerase activity using the TRAP real-time PCR assay. We observed a significantly elevated telomerase activity in the testicular tissue of MD-LPD males when compared to NPD and LPD males. (Fig. 5F; *P* < 0.01).

**Figure 6:**
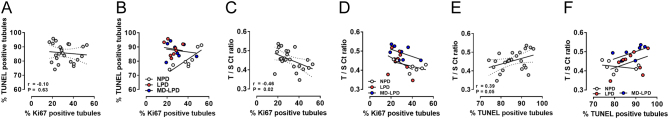
Impact of NPD, LPD and MD-LPD on correlation between the percentage of Ki67 positive stained tubules to percentage of TUNEL positive tubules in all males (A) and individual dietary groups (B); correlation between the percentage of Ki67 positive stained tubules and T/S ratio in all males (C) and individual dietary groups (D) and correlation between percentage of TUNEL positive tubules and T/S ration in all males (E) and individual dietary groups (F). Data are mean ± s.e.m. *n* = 8 males per dietary group. Data were analysed by Pearsons correlation.

Our final analyses examined the association of testicular telomere length (T/S ratio), apoptosis (TUNEL staining) and proliferation (Ki67 staining). We observed no association between TUNEL staining and levels of Ki67 staining when all males were analysed together (Fig. 6A). However, NPD males displayed a significant positive association (Fig. 6B; *r* = 0.83, *P* = 0.01) which was not present within either LPD or MD-LPD males. Analysis of the association between telomere length and Ki67 staining revealed no association, either when all males were analysed together or as separate diets. Finally, while a positive association was seen between TUNEL staining and telomere length when all males were analysed together (Fig. 6E; *r* = 0.4, *P* = 0.05), no association was observed when data for individual diets were analysed separately (Fig. 6F).

## Discussion

There is increasing evidence that poor paternal diet at the time of conception impacts significantly on long-term offspring health. Underlying this association has been the development of a range of animal models which have supported detailed investigation into the mechanisms driving the paternal programming of offspring ill-health. In our current study, we observe that male mice fed either a low protein diet (LPD) or LPD supplemented with methyl-donors (MD-LPD) display significantly altered levels of serum fatty acids, testicular morphology, apoptosis, cellular proliferation and telomere length. However, these diets did not alter fundamental aspects of male reproductive fitness such as testosterone levels, litter size or late gestation fetal weight. These observations show the impact of sub-optimal paternal diet on male physiology and testicular morphology which may provide new insight into the mechanisms linking paternal diet with sperm quality and the programming of offspring ill-health.

Studies in rodents have shown that diets low in protein result in increased energy intake and energy expenditure associated with no change in fat mass (White *et al*. 2000, Aparecida de Franca *et al*. 2009, Laeger *et al*. 2014). However, diets deficient in methionine and/or cysteine have been shown to decrease food intake (Plaisance *et al*. 2010), increase energy expenditure (Wanders *et al*. 2015), have beneficial effects on glucose/insulin sensitivity and cardiac function and increase longevity in animal studies (Ables & Johnson 2017). In humans, a methionine restricted diet has also been shown to result in weight loss, decreased adiposity and improved insulin sensitivity (Plaisance *et al*. 2011). Furthermore, epidemiological studies have identified significant positive correlations between total plasma cysteine levels, fat mass and obesity (El-Khairy *et al*. 1999, Elshorbagy *et al*. 2008). Interestingly, studies have shown that, in fasted obese individuals, levels of serum folate are lower, while levels in red blood cells are higher than in non-obese people (Bird *et al*. 2015). These studies suggest that elevated levels of folate, methionine and/or cysteine, or an impairment in their metabolism, may associate with obesity, increased circulating lipid profiles and insulin insensitivity.

Underlying the physiological changes observed in our dietary manipulated males may be alterations in hepatic lipid metabolism and fatty acid synthesis. An untargeted analysis of serum fatty acids in our males revealed significant changes across a range of medium (6–12 carbon) and long (13–21 carbon) fatty acids. MD-LPD males displayed significantly higher levels of serum saturated fatty acids when compared to LPD males. However, levels of monounsaturated and polyunsaturated fatty acids, including the essential polyunsaturated omega-6 fatty acid linoleic acid (C18:2), were significantly lower in MD-LPD when compared to NPD and LPD males. In contrast, LPD males displayed a fatty acid profile opposite to that of MD-LPD males with significantly lower levels of the saturated capric acid, myristic acid, palmitic acid and heptadecanoic acid, and increased levels of stearic acid, arachidonic acid and lignoceric acid when compared to NPD and/or MD-LPD males. One possible explanation for the different fatty acid profiles of LPD and MD-LPD males is the selective tissue incorporation of individual fatty acid species into other tissues and/or their mobilisation from the liver. For instance, increased plasma stearic acid, arachidonic acid and lignoceric acid concentration could be, in part, the result of selective adaptation to hepatic fatty acid synthesis (Burdge *et al*. 1994), which may increase the availability of these fatty acids to other tissues including the testis. LPD males also displayed significantly elevated levels of arachidonic acid when compared to NPD males. Arachidonic acid is a precursor of the major classes of inflammatory and chemotactic lipids, eicosanoids (Sonnweber *et al*. 2018). Therefore, elevated levels within the serum may indicate underlying perturbations in hepatic metabolism (Bozza *et al*. 2011), enhanced inflammatory status (Dennis & Norris 2015) and/or even cardiovascular disease (Merched *et al*. 2008) in response to the LPD. However, further studies are needed to define the precise metabolic disturbances occurring in our mice and their underlying causes.

Interestingly, we observed that the largest differences in fatty acid profile occurred between LPD and MD-LPD fed males, suggesting supplementation with methyl donors, separate to the effects of the LPD, modified fatty acid profile. In rats, a 2% LPD has been shown to decrease levels of hepatic phospholipids while increasing levels of triacylglycerols, unesterified cholesterol, and cholesteryl esters (Meghelli-Bouchenak *et al*. 1989). Similarly, an 8% LPD resulted in glucose and insulin intolerance, increased serum adiponectin levels and lowered very low density lipoprotein (VLDL) levels in rats (Kang *et al*. 2011). In contrast, a high protein low carbohydrate diet fed to patients with non-alcoholic fatty liver disease (NAFLD) reduced fasting glucose levels, total low density lipoprotein (LDL), VLDL and triglycerides and increased high density lipoprotein (HDL) (Bezerra Duarte *et al*. 2014). Additionally, diets low in carbohydrate reduce triglyceride and HDL levels in humans (Hu *et al*. 2012), while in gerbils, a high carbohydrate diet results in histopathological and metabolic abnormalities characteristic of nonalcoholic steatohepatitis (NASH) (Semiane *et al*. 2017). Diets deficient in 1-carbon metabolites such as folate, methionine and choline have also been shown to impair lipid metabolism (Koteish & Diehl 2001, Henkel *et al*. 2012) resulting in reduced flux through the phosphatidylethanolamine *N-*methyltransferase (PEMT) pathway (Chew *et al*. 2011). Furthermore, significant increases in the expression of lipid biosynthesis genes such as elongation of very long chain fatty acids protein 2 (*Elovl3*), long-chain-fatty-acid-CoA ligase 1 (*Acsl1*) and ATP-citrate synthase (*Acly*) have been reported in folate deficient mice (Champier *et al*. 2012). Interestingly, excessive folate intake, in conjunction with high fat diet, also impairs hepatic fatty acid oxidation promoting hepatic lipid accumulation in rats (Burdge *et al*. 2009). These data suggest deficiencies and/or excesses in 1-carbon metabolites may reduce hepatic phospholipid biosynthesis (Walker *et al*. 2011), potentially through changes in the epigenetic regulation of hepatic function. Indeed, hypermethylation of the promoter for *Fads2* in mice has been shown to result in increased levels of the unsaturated long-chain arachidonic and docosahexanoic acid (Devlin *et al*. 2007). In addition, other genes involved in fatty acid elongation, such as Elovl2, have been shown to be epigenetically regulated with age (Bacalini *et al*. 2017), identifying links between epigenetic status and metabolic health.

In LPD fed males, we also observed significant reductions in the levels of fatty acids with 17 carbons or less, but increased levels of fatty acids with 18 carbons or more. These observations might indicate altered flux through the fatty acid synthesis pathway in response to the LPD, with a shift from *de novo* lipogenesis to increased elongation of existing palmitic acid. Conversely, MD-LPD males showed a profile indicative of perturbed desaturation as evidenced by higher levels of saturated fatty acids. Indeed, LPD males displayed decreased levels of C17:0, while MD-LPD males displayed elevated levels. Recently, studies have shown negative associations between circulating levels of the odd chain fatty acids, pentadecanoic acid (C15:0) and heptadecanoic acid (C17:0), with metabolic disease risk (Jenkins *et al*. 2017*b*). Here, increased intake of dairy products is associated with elevated C15:0 and C17:0, as rumen bacteria are believed to be the main producers of these odd chain fatty acids. However, in our study, the main source of fat was in the form of corn oil which contains a trace (0.1%) of C17:0. As such, the altered levels of serum C17:0 seen in the LPD and MD-LPD males is unlikely due to their diet. Studies in mice have shown that shown that C17:0 can be made endogenously through the action of 2-hydroxyacyl-CoA lyase 1 (Hacl1) and the alpha oxidation of phytanic acid (Jenkins *et al*. 2017*a*). However, as we did not assess the levels of alpha-oxidation of phytanic acid or the activity of hepatic Hacl1 in our males, we cannot comment on the mechanisms underlying the differences in c17:0 observed in the LPD and MD-LPD males.

While no differences in the hepatic expression of Acyl-CoA desaturase 1 (*Scd1*), the fatty acid binding proteins 1 and 3 (*Fabp1/3*) or Acyl-CoA 6-desaturase (*Fads2*) were determined, we did observe significant decreases in the ratio of linoleic (C18:2) to arachidonic (C20:4) acid between NPD and LPD males, an indicator of increased activity of the desaturases Fads1/2 and/or elongase Elovl5. Furthermore, we observed an increased C18:0 to C18:1 ratio in MD-LPD when compared to NPD males, suggestive of decreased activity of the Acyl-CoA desaturase 2 (*Scd2*). In pigs, feeding of a low protein diet has been shown to elevate the expression of fatty acid desaturase 1 (FADS1) (Madeira *et al*. 2016), while in rats fed a high carbohydrate diet, increased hepatic expression of *Fads2*, *Scd1/2* and *Elovl5/6* were observed (Drag *et al*. 2017). While these observations may explain, in part, the differences in levels of saturated and unsaturated fatty acids observed between groups, further, more detailed mechanistic and epigenetic analyses would be required determine fully define the pathways regulating fatty acid profiles in in response to diet.

A second key finding of our study was that paternal diet impacted significantly on testicular morphology. LPD males displayed significantly increased mean seminiferous tubule size, while seminiferous tubule morphology in MD-LPD males was more similar to that of NPD males. Underlying changes in tubule morphology are a range of processes including inflammation (Loveland *et al*. 2017), oxidative damage (Turner & Lysiak 2008) hormone imbalance (Appasamy *et al*. 2007) and apoptosis (Theas 2018). As LPD males possessed fewer tubules with apoptotic cells and a trend toward increased rates of proliferation when compared to NPD males, we focused on the gene expression profiles of central pro and anti-apoptotic genes. Apoptosis is an integral part of the tightly regulated process of spermatogenesis, ensuring an appropriate number of germ cells per sertoli cell (Aitken *et al*. 2011). We observed no differences in gene expression patterns between NPD and LPD males. In contrast, MD-LPD testes displayed decreased expression of the pro-apoptotic genes *Bax, Csap1* and *Fas* when compared to NPD males and a higher expression of the testis-specific chromatin gene *Brdt*. Treatment of mice with an anti-Fas neutralising antibody prevents testicular germ cell death (Lee *et al*. 1997). Conversely, androgen withdrawal in rats upregulates testicular germ cell apoptosis and the expression of Fas (Woolveridge *et al*. 1999). Similarly, the role of the Bcl-2 family, which includes Bad and Bax, have been well studied and shown to be involved in spontaneous apoptosis in normal human testes (Oldereid *et al*. 2001). Studies have shown that methionine restriction in mice can reduce rates of proliferation and increase the rates of apoptosis in mammary tumours (Hens *et al*. 2016). Conversely, choline and betaine supplementation have been shown to reduce cancer risk in humans (Sun *et al*. 2016), while in mice, maternal choline supplementation during gestation reduces rates of placental apoptosis (King *et al*. 2019). Interestingly, tubules from LPD males displayed a trend toward a higher proportion staining positive for the proliferation marker Ki67. Ki67 is recognised as an established marker of active cell division and widely used in analysis of testicular morphology and spermatogenic cell proliferation (Zhao *et al*. 2018). Therefore, our data indicate that the changes in tubule morphology observed in LPD males may be influenced more by increased rates of cellular proliferation as apposed to increased rates of cell loss. However, expression analysis of multiple seminiferous tubule cell-specific markers revealed comparable levels between NPD and LPD males, suggesting that significant changes (either gain or loss) in specific cell populations within the tubule may not be completely responsible for the changes in morphology observed. Further studies to explore other pathways known to regulate seminiferous tubule morphology (e.g. androgen signalling, inflammation) are required to define in detail the mechanisms linking paternal diet with testis morphology.

Interestingly, while we observed significant changes in testicular morphology, we did not observe any impairment in fundamental reproductive fitness in any of our males. These observations are in line with our previous studies showing no effect of LPD on testosterone levels, testis weight, sperm production, numbers of embryos collected or mean litter size (Watkins & Sinclair 2014, Watkins *et al*. 2017, 2018). These observations suggest that aspects of semen quality such as sperm morphology or motility are unaltered in response to the LPD or MD-LPD, in agreement with our previous observation that sperm from LPD males show no impairment in capacitation profile (Watkins *et al*. 2018). However, we cannot rule out that the LPD and MD-LPD may affect other aspects of sperm quality, such as epigenetic status, which were not assessed in detail in this study. Indeed, recent mouse studies showing alterations in specific sperm RNA populations can transit paternal phenotypic characteristics to subsequent generations (Chen *et al*. 2016, Zhang *et al*. 2018) support the hypothesis that conventional semen analysis may not reveal subtle change in sperm quality which can influence post-fertilisation development and offspring health. Therefore, more detailed epigenetic analysis of the sperm from LPD and MD-LPD males, as well as analysis of the seminal plasma (Morgan & Watkins 2019) is warranted. Furthermore, it would be of interest to examine the effect of a methyl donor-supplemented NPD on male reproductive fitness, as some studies report altered offspring cognitive function in response to paternal methyl donor-rich diets (Ryan *et al*. 2018).

Our final observation was that MD-LPD testes displayed an increased rate of telomerase activity which coincided with a reduced telomere length, as indicated by a significantly increased T/S ratio. In the mature mouse testis, the telomerase protein (Tert) has been shown to be expressed in spermatogonia, early spermatocytes and elongating spermatids but not in round spermatids or pachytene spermatocytes (Tanemura *et al*. 2005). Interestingly, analysis of telomere length across the different cells of the spermatogenic cycle has revealed elongating spermatids to have the longest length (Tanemura *et al*. 2005). However, in the human (Izadyar *et al*. 2011), mouse (Martin-Rivera *et al*. 1998) and rat (Ravindranath *et al*. 1997), the highest rates of telomerase activity have been reported in the spermatogonial stem cells. As such, the cells with the longest telomere length have the lowest levels of telomerase activity (Tanemura *et al*. 2005). As in the MD-LPD males, the apparent discrepancy between telomere length and telomerase activity may reflect an inability, or over compensation, of telomerase in the testicular germ cells to elongate premeiotic spermatocytes appropriately, resulting in shorter telomere lengths overall (Ozturk 2015).

We have previously shown that a paternal LPD impacts significantly on sperm epigenetic status, embryo development, fetal growth and adult cardiovascular and metabolic health (Watkins & Sinclair 2014, Watkins *et al*. 2017, 2018). Our current study demonstrates that underlying these changes may be significant changes in testicular morphology. In contrast, we observe that supplementation of the LPD with specific methyl donors results in a morphology more similar to that of the control diet fed males, changes which may have be driven by altered expression profiles of central regulators of apoptosis and cellular proliferation. However, our current study demonstrates that methyl-donor supplementation also alters serum fatty acid profiles significantly, increasing levels of harmful saturated fatty acids and decreasing beneficial unsaturated fatty acids. Therefore, it is essential that further studies are conducted to determine the precise impact of methyl-donor supplementation on sperm quality, fetal development and long-term offspring health. It would also be of significant interest to relate the serum fatty acid profiles of our males to profiles in the testes, sperm and seminal plasma. Such studies are necessary to define how the intake of such dietary supplements may affect male reproductive health and the health of his offspring.

## Declaration of interest

The authors declare that there is no conflict of interest that could be perceived as prejudicing the impartiality of the research reported.

## Funding

This work is supported by funding from the BBSRC (BB/R003556/1) to A J W. I A is supported by the Commonwealth Scholarship Commission, UK, under grant reference GHCS-2016-146.

## Author contribution statement

H L M, I A, H R G and A J W performed conception and design of the experiments. H L M, I A, N E, C R, H R G and A J W collected and analysed the data. H L M, I A, N E, C R, H R G and A J W performed data interpretation. H L M, I A, H R G and A J W drafted the article. H L M, I A, H R G and A J W revised the article critically for intellectual content.
